# Behavior-psychological linkages: how health literacy enhances life satisfaction in Chinese university students through the mediation of persistent exercise and emotional management ability

**DOI:** 10.3389/fpsyg.2026.1706872

**Published:** 2026-01-29

**Authors:** Hao-jie Zuo, Ting-ting Liu, Ding-you Zhang, Yu-cai Xu, Hu Lou, Shan-shan Han, Shi-hai Yu, Bo Li

**Affiliations:** 1 Institute of Sports Science, Nantong University, Nantong, China; 2 Institute of Mathematics and Statistics, Nantong University, Nantong, China

**Keywords:** chain mediation effect, emotional management ability, health literacy, life satisfaction, persistent exercise, university students

## Abstract

**Objective:**

This study investigates the associative mechanism between health literacy and life satisfaction among university students in China, specifically examining the chain mediation pathway involving persistent exercise and emotional management ability. Health Literacy is understood as the capacity of an individual to access, comprehend, appraise, and utilize relevant health information to promote and maintain well-being proactively. Complementing this is the concept of Life Satisfaction, which denotes an individual’s subjective assessment of their overall circumstances and quality of life. Furthermore, Persistent Exercise refers to the sustained and regular engagement in physical activity necessary for long-term physical conditioning. Finally, Emotional Management Ability—often framed as emotional intelligence—is the aptitude for an individual to effectively recognize, understand, modulate, and appropriately deploy their affective states. It explores the chain-mediated roles of persistent exercise and emotional management ability. Considering persistent exercise as a behavioral factor and emotional management ability as a psychological factor, this research aims to provide a theoretical basis for enhancing the quality of life of university students by investigating the chain-mediated effects of these factors.

**Methods:**

This research constitutes a cross-sectional investigation. Data collection was standardized across administrative classes in September 2024, facilitated through the Wenjuanxing survey platform. A multi-stage stratified cluster sampling method was employed to select 16,395 university students across five provinces in China. The sample size was determined through proportionate allocation, benchmarked against the total national population of university students. A minimum sample size of 45 participants was required for each stratum—subgroups defined by gender and academic year/grade level—to achieve the necessary statistical power for the analysis. Measurements were operationalized using the Health Literacy Scale (HLS-SF9), the Satisfaction with Life Scale (SWLS), a scale for measuring exercise adherence, and the Emotional Intelligence Scale (EIS). Statistical analyses were conducted utilizing SPSS Version 26.0, the PROCESS macro (Version 4.2), Microsoft Excel, and MATLAB. The methods employed included descriptive statistics, correlation analysis, and regression analysis.

**Results:**

Health literacy was significantly positively correlated with life satisfaction (*β* = 0.184, *p* < 0.001). Persistent exercise and emotional management ability appeared to act as chain mediators in the relationship between health literacy and life satisfaction, with a total effect of 0.506 (95% *CI* [0.486, 0.525]), a total direct effect of 0.183 (95% *CI* [0.164, 0.203]), and a total indirect effect of 0.322 (95% *CI* [0.306, 0.338]). Specifically, health literacy demonstrated a positive association with life satisfaction (*β* = 0.090, *p* < 0.001; *β* = 0.520, *p* < 0.001), as it both enhanced persistent exercise (*β* = 0.952, *p* < 0.001) and improved emotional management ability (*β* = 0.277, *p* < 0.001). The chain-mediated pathway “Health literacy → Persistent exercise → Emotional management ability → Life satisfaction” had an effect of 0.092 (95% CI [0.085, 0.099]).

**Conclusion:**

This study revealed a significant association between health literacy and life satisfaction among Chinese university students. Furthermore, persistent exercise and emotional management ability were found to be potentially associated with both life satisfaction and health literacy, suggesting possible indirect pathways.

## Introduction

Amidst China’s economic and social transition toward high-quality development, residents’ life satisfaction has become a key indicator for measuring population well-being. As a cognitive dimension of subjective well-being (Kushlev et al., 2020), life satisfaction significantly influences the psychological health development of university students (Preetz et al., 2021). As a crucial force in the nation’s future, the life satisfaction of university students reflects the quality of higher education and the effectiveness of youth development policy implementation. It is linked to the progress of the “talent-strong nation” strategy. The “Fourteenth Five-Year Plan” (National Development and Reform Commission of the People's Republic of China, 2021) explicitly sets development goals, providing a systematic framework for improving university student life satisfaction. Related guidance from the Ministry of Education emphasizes attention to student development needs and improvements in campus life satisfaction (Ministry of Education of the People's Republic of China, 2018), fully demonstrating the nation’s concern for the quality of life of university student cohorts. Therefore, a thorough investigation into the factors influencing life satisfaction among university students is of significant theoretical and practical importance. However, the life satisfaction of university students currently faces numerous challenges. Research suggests that lower life satisfaction can significantly affect the psychological health of university students (Diener and Seligman, 2002), leading to decreased academic performance (Lyubomirsky et al., 2005) and negatively impacting physical health (Steptoe et al., 2009). Notably, these effects may extend to career development after graduation, with far-reaching impacts on the long-term development of university students (Li et al., 2015). As an essential cohort driving social progress, life satisfaction among university students has garnered widespread attention, emerging as a priority for both academic and social sectors (Wilcox and Nordstokke, 2019; Rogowska et al., 2021). While existing research has preliminarily explored the relationships between health literacy, persistent exercise, emotional management ability, and life satisfaction, it often lacks an integrated theoretical framework to explain the complex mechanisms underlying these factors comprehensively (Nutbeam and Lloyd, 2021; Gross, 2015). Current studies usually focus on isolated investigations or simple linear relationships without adequately considering the potential interactions and multiple mediating effects among these factors (Berkman et al., 2011; Mikkelsen et al., 2017). This study aims to address the research gap by developing an integrated theoretical framework that combines the Health Belief Model (HBM) (Von Wagner et al., 2009), Social Cognitive Theory (SCT) (Rovniak et al., 2002), and the Biopsychosocial Model (BPSM) (Hinostroza and Mahr, 2025). This research provides an in-depth examination of the relationship between health literacy and life satisfaction among Chinese university students, specifically through the mediating roles of persistent exercise and emotional management ability. A particular emphasis will be placed on the points of convergence and complementarity between these different theories to overcome the limitations of existing research. Concurrently, within the cultural context of this study, the Chinese traditional philosophy, which emphasizes the unity of body and mind (Kohn, 2008), offers profound indigenous insight into how physiological well-being (i.e., persistent exercise) and psychological welfare (i.e., emotional management ability) collectively influence overall life satisfaction. This holistic perspective naturally aligns with the multidimensional interplay emphasized by the Western BPS Model, while simultaneously providing a deeper cultural foundation for behavioral intervention guided by HBM (persistent exercise) and self-efficacy conceptualized by SCT (emotional management ability), thus offering a superior explanation for the complex experiences of Chinese university students.

Health literacy refers to an individual’s ability to obtain, understand, and apply health information (Arriaga et al., 2022), and the level of this ability has a direct association with personal health status (Berkman et al., 2011). Research indicates that lower levels of health literacy are significantly related to increased hospitalization rates and elevated use of accident and emergency services, potentially contributing to risks to an individual’s physical and mental health (Bernstein et al., 2020). Due to their selection through the gaokao examination and subsequent cultivation through higher education, Chinese university students generally exhibit higher levels of health literacy than the general population (Quenzel et al., 2015). Furthermore, a significant correlation exists between physical well-being (including health literacy, health level, activities of daily living, and risk factors) and life satisfaction among university students (Kaučič et al., 2019), with specific abilities directly impacting health-related decision-making. The HBM suggests that improved health cognition is closely related to adopting corresponding behaviors, which positively correlate with maintaining physical and mental health (Von Wagner et al., 2009). Research has demonstrated a positive correlation between health literacy and health-related quality of life (Liu et al., 2021), which is also strongly correlated with improvements in both physical and mental health (Von Wagner et al., 2009) and may potentially influence life satisfaction (Naar, 2012). Compared to other groups, university students may be more focused on health, and this can be achieved through health literacy, potentially leading to improvements in both physical and mental well-being, which in turn influences their life satisfaction.

Persistent exercise refers to regular physical activity undertaken by an individual to enhance their physical and mental well-being over an extended period (Chen et al., 2020a). SCT posits that individual behavior is determined by the interaction of cognitive factors, behavioral performance, and environmental influences (Rovniak et al., 2002). When accessing health information, such as through fitness apps, university students are encouraged to engage in more physical exercise (Arriaga et al., 2022) and enhance their knowledge and self-efficacy in this area. This empowers them to overcome barriers to exercise and maintain long-term exercise behavior (Bandura, 1997). The characteristic of persistence that individuals exhibit about exercise is often associated with positive goal orientation (Jung and Brawley, 2010), potentially alleviating psychological distress (Schuch et al., 2016). Through regular physical exercise, university students may experience physical and mental improvements that strengthen their self-efficacy (Li et al., 2024). Environmental factors, such as the accessibility of campus sports facilities and peer support, are associated with maintaining exercise behavior, potentially increasing feelings of self-accomplishment and control over their lives, thereby influencing their life satisfaction (Diener et al., 1999).

Positive psychological states play a crucial role in coping with stressful situations. Individuals with strong emotional management ability can significantly reduce the occurrence of negative emotions, such as depression and anxiety (Naragon-Gainey et al., 2017). Health literacy may also contribute to proactive behaviors in emotional management when processing information (Mõttus et al., 2014). Individuals with low health literacy may be more susceptible to emotional regulation disorders, such as anxiety and depression. Interventions targeting health literacy can potentially strengthen their emotional management ability (Berkman et al., 2011). As university students navigate a critical stage in their development while facing multiple pressures from academics, employment prospects, and interpersonal relationships, they may be prone to experiencing negative emotions, such as anger and tension (Arsenio and Loria, 2014). If university students with inadequate emotional management ability struggle to cope with these negative emotions, accumulated emotional distress can potentially reduce their life satisfaction (Gross and John, 2003). Research has shown that long-term regular exercise may improve cardiovascular and immune function (Bennett et al., 2017). It also regulates the neuroendocrine system, promoting the secretion of neurotransmitters such as endorphins and serotonin, which may potentially alleviate stress through these regulatory mechanisms (Rodrigues et al., 2020). Enhancing positive emotions and increased emotional stability may also be related to these physiological mechanisms (Rodrigues et al., 2021; Mu et al., 2024). University students demonstrating higher psychological resilience under academic and social pressures may experience direct physiological effects on their psychological state.

The relationship between health literacy and life satisfaction has emerged as a key area of research in public health and psychology. Existing research has demonstrated a positive relationship between health literacy and an individual’s life satisfaction (Naar, 2012; Bakkeli, 2021). At the cognitive level, individuals with higher health literacy are better equipped to access, comprehend, and evaluate health information, thereby enabling them to make more informed health decisions (Berkman et al., 2011; Arriaga et al., 2022). This sense of control over health information often fosters greater confidence in facing health challenges, thereby reducing anxiety stemming from uncertainty (Diener et al., 1999) and subsequently enhancing overall well-being. At the behavioral level, improved health literacy is frequently associated with the adoption and maintenance of positive health behaviors, such as persistent exercise, healthy eating habits, and abstaining from smoking and excessive alcohol consumption (Aaby et al., 2017). Multiple studies have shown that individuals with high health literacy are more likely to engage in physical activity and sustain it over the long term (He et al., 2025; Ayaz-Alkaya and Kulakçı-Altıntaş, 2021). These healthy behaviors not only improve an individual’s physical health (Liu et al., 2024) but also indirectly elevate life satisfaction by strengthening self-efficacy and feelings of accomplishment (Li et al., 2024; Dai and Yao, 2012). However, current research lacks in-depth investigation into the specific mechanisms through which health literacy impacts life satisfaction via particular health behaviors, such as “persistent exercise,” particularly within the context of university students, whose unique learning and living environment may imbue “persistent exercise” with a more complex role. Furthermore, the impact of health literacy on mental health, especially emotional management ability, has also gained increasing attention (Mõttus et al., 2014). Individuals with high health literacy, when processing information, are more proactive in employing emotion regulation strategies, effectively addressing negative emotions, and consequently reducing the incidence of depression and anxiety (Mõttus et al., 2014; Mann, 2004). Enhanced emotional management ability enables individuals to better adapt to life stressors, maintain a positive psychological state, and, consequently, directly increase life satisfaction (Gross and John, 2003; Rodrigues et al., 2021).

It is essential to note that, while the majority of studies support a positive association between health literacy and life satisfaction, some studies have reported inconsistent or contradictory findings. Certain cross-sectional studies, although revealing correlations, have failed to establish the direction of causality (Kaučič et al., 2019). Furthermore, the strength and specific pathways through which health literacy impacts life satisfaction may vary across different cultural backgrounds and socioeconomic conditions (Duong et al., 2017; Liu et al., 2020). This may be attributed to the heterogeneity of study participants (e.g., age, educational attainment, health status, etc.), differences in measurement tools, and limitations in the selection of mediating variables. For instance, some studies may have focused solely on a single mediating pathway (e.g., concentrating on health behaviors or psychological states alone), overlooking the complexity of multi-pathway interactions, which could potentially underestimate the comprehensive effects of health literacy (Berkman et al., 2011). Although existing research has preliminarily established a link between health literacy and life satisfaction (Berkman et al., 2011; Van Der Heide et al., 2013), there is a lack of sufficient exploration regarding the integrated mechanisms through which health literacy influences life satisfaction via behavioral pathways (particularly “persistent exercise,” a specific behavior highly relevant to university students) and psychological pathways (“emotional management ability,” a crucial psychological resource), especially the chain mediating mechanisms, particularly for the specific population of Chinese university students. Despite some studies mentioning the relationships between health literacy, health behaviors, and psychological states (Nutbeam, 2000; Paasche-Orlow and Wolf, 2007), there is a lack of systematic empirical testing of the multiple, sequential chain mediating effects among these variables, which fail to clearly elucidate the internal transmission mechanisms. In addition, there has been an insufficient in-depth analysis of the differential contributions of various components of health literacy (e.g., information acquisition ability, comprehension ability, application ability) in influencing life satisfaction, as well as their roles in multiple mediation models.

Therefore, based on the aforementioned research background and the identified gaps in the literature, this study aims to construct a multiple-chain mediation model to explore the relationships among health literacy, persistent exercise, emotional management ability, and life satisfaction among Chinese university students. The core research questions of this study are: (1) Does health literacy directly influence the life satisfaction of Chinese university students? (2) Does health literacy indirectly improve the life satisfaction of Chinese university students through the mediating role of persistent exercise? (3) Does health literacy indirectly improve the life satisfaction of Chinese university students through the mediating role of emotional management ability? (4) Does health literacy jointly improve the life satisfaction of Chinese university students through a chain mediating pathway involving persistent exercise and emotional management ability? The novelty of this study lies in its integration of the HBM, SCT, and BPSM to construct a more comprehensive theoretical framework. Specifically, the HBM explains how health literacy influences health beliefs and behaviors (Von Wagner et al., 2009), the SCT provides insights into the formation of persistent exercise behavior (Rovniak et al., 2002), and the BPSM emphasizes the interplay of biological, psychological, and social factors (Hinostroza and Mahr, 2025), thereby providing a more holistic perspective on the role of emotional management ability in the relationship between health literacy and life satisfaction. By integrating these theories, this study transcends the limitations of single-theory approaches in existing research. It aims to offer a more comprehensive understanding of the intricate relationship between health literacy and life satisfaction. Through chain mediation analysis, this study innovatively reveals the dual influence mechanisms of health literacy on university students’ life satisfaction, mediated by persistent exercise (behavioral pathway) and emotional management ability (psychological pathway).

**Figure 1:**
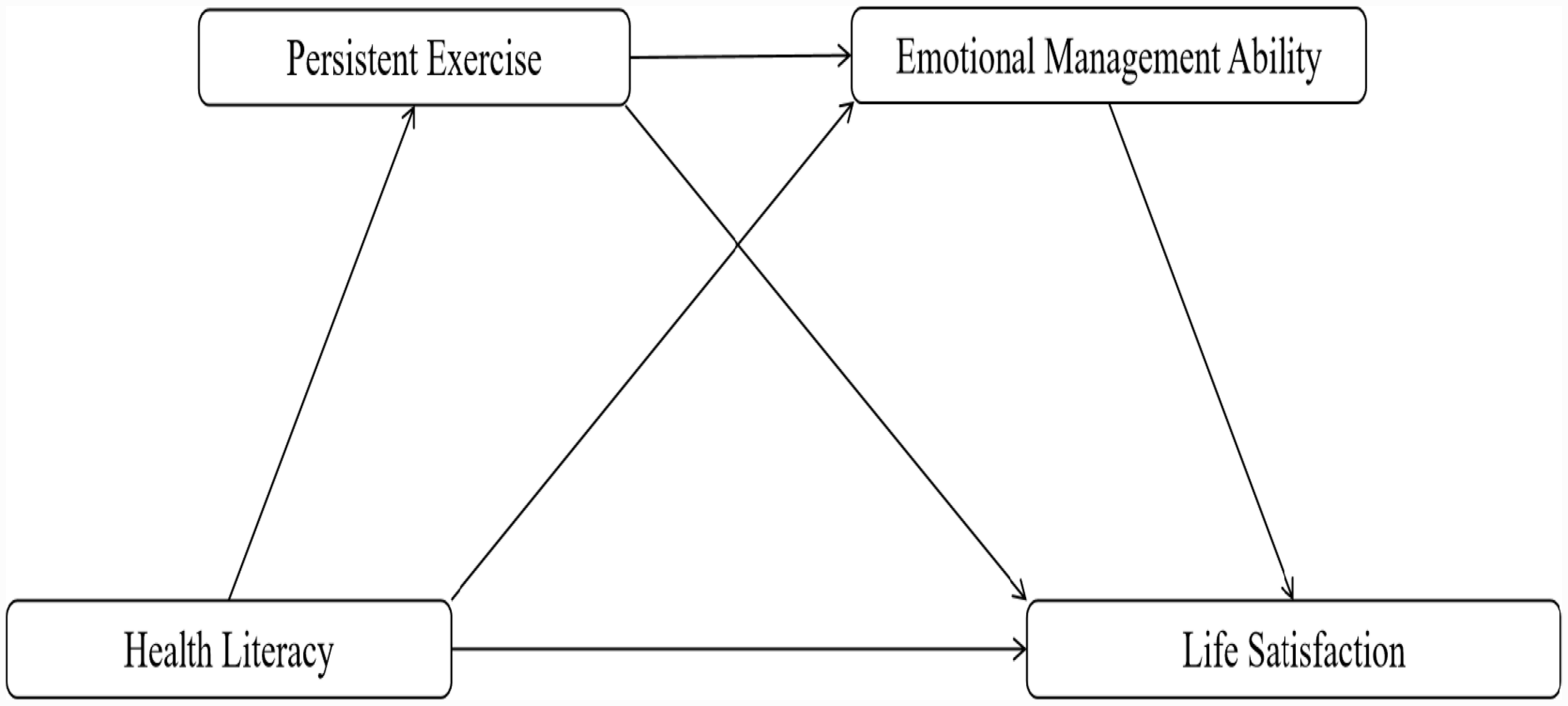
Hypothetical model diagram.

In summary, this study aims to thoroughly investigate the various mechanisms by which health literacy affects the life satisfaction of Chinese university students and to offer practical guidance for enhancing their psychological well-being and overall welfare. The findings of this research will contribute to a more comprehensive understanding of the crucial roles played by persistent exercise and emotional management ability in this process, providing a scientific basis for developing more effective intervention strategies. This study not only enriches the research on health literacy but also provides theoretical support and practical direction for enhancing university students’ life satisfaction and fostering their comprehensive development. The theoretical hypotheses proposed by this study, along with the corresponding conceptual framework, are illustrated in Figure 1.

*H1*: Health literacy is positively associated with life satisfaction in Chinese university students.

*H2*: Persistent exercise mediates the positive association between health literacy and life satisfaction in Chinese university students.

*H3*: Emotional management ability mediates the positive association between health literacy and life satisfaction in Chinese university students.

*H4*: The positive association between health literacy and life satisfaction in Chinese university students is serially mediated by persistent exercise and emotional management ability.

## Methods

### Study design

This investigation constitutes a nationwide, cross-sectional survey study designed to explore the relationships among health Literacy, persistent exercise, emotional management ability, and life satisfaction. Data were collected online via the Wenjuanxing platform during September 2024.

#### Target population

The target population for this study comprises full-time enrolled students attending institutions of higher education within Mainland China.

#### Inclusion criteria

Participants were included if they met all of the following criteria: (1) Currently enrolled students at an institution of higher education registered with the Ministry of Education (encompassing both undergraduate and vocational/junior college levels). (2) Aged between 15 and 30 years, inclusive. (3) Completed the questionnaire in its entirety with no apparent logical inconsistencies.

#### Exclusion criteria

Questionnaires were excluded if any of the following conditions were met. (1) The respondent’s institution name was illegible or could not be verified. (2) The respondent’s age fell outside the 15–30 year range. (3) The completion pattern exhibited evidence of invalid response behavior, specifically, 21 or more consecutive items answered identically (straight-lining). (4) Data points were identified as outliers based on data cleaning guidelines derived from the International Physical Activity Questionnaire (IPAQ) principles. (5) Completion time fell within the extreme 5% tails of the total time distribution (i.e., excessively short or excessively long completion times), to rule out data resulting from careless responses or undue procrastination.

#### Sample size determination rationale

The determination of the required sample size was not based solely on a singular formula derived from overall population proportions, but rather centered on ensuring adequate analytical power for key demographic subgroups (strata) across the entire nation (Kish, 1965; Cohen, 2013). The calculation process commenced by stratifying the primary analysis by gender (male/female) and year of study (University years 1–4). Following established methodological recommendations, a minimum target sample size of 45 participants per subgroup was set to guarantee fundamental statistical power for each resulting stratum. Given the multi-stage stratified sampling design, which mandated selecting samples from three cities representing differing economic development levels within each province, the total number of subgroups requiring coverage per province was 2 (Gender) × 4 (Year of Study) × 3 (Sampling Locations) = 24. This resulted in a planned provincial sample size of 24 subgroups × 45 participants/subgroup, 1,080 participants. Extrapolating this to cover all 31 Provinces, Autonomous Regions, and Municipalities in mainland China (excluding Hong Kong, Macau, and Taiwan), the projected national total sample size was calculated to be 31 × 1,080 = 33,480 participants. This framework was designed specifically to guarantee the representativeness of crucial subpopulations at both the national and provincial strata. In terms of data quality control, rigorous screening was applied to ensure questionnaire completeness and internal consistency, resulting in the exclusion of invalid responses. Ultimately, 36,756 questionnaires were returned, successfully surpassing the initial projection.

The final sample incorporated into the analysis comprised 16,395 valid questionnaires collected from students in selected higher education institutions across Jiangsu, the Northeast region (Liaoning, Jilin, and Heilongjiang), Henan, Hubei, and Guangdong. This achieved sample size is deemed entirely sufficient to meet the statistical power requirements necessary for the mediation effect modeling central to this study (MacKinnon et al., 2004; Kline, 2023). Geographically, these selected areas represent four major national divisions—East China, North China, Central China, and South China—thereby enabling the capture of regional variations in higher education sports development influenced by factors such as economic maturity, climatic conditions, and cultural traditions. The precise distribution of the sample across these regions is detailed in Table 1.

**Table 1:** Sample characteristics.

Variable	*n*	%
Gender		
Male	6,043	36.86
Female	10,352	63.14
Grade		
Freshmen	10,170	62.03
Sophomores	5,112	31.18
Juniors	984	6.00
Seniors	129	0.79
Smoking behavior		
Never smoked	15,017	91.59
Occasional smoking	1,026	6.26
Frequent smoking	352	2.15
Drinking behavior		
Never drank	7,943	48.45
Drank without getting drunk	8,098	49.39
Drank and got drunk	354	2.16
Total	16,395	100

### Sampling methodology

To ensure the national representativeness and the necessary geographical and economic diversity of the sample, this study employed a multi-stage stratified cluster sampling methodology.

#### Selection of sampling locations

To ensure comprehensive coverage of diverse regional characteristics, the selection of sampling sites considered factors such as geographical location and economic development levels. Firstly, the entire country was divided into provinces, autonomous regions, and municipalities directly under the central government, according to the administrative divisions of the National Bureau of Statistics of China. Subsequently, within each province/autonomous region/municipality, stratification was conducted based on economic development level and geographical location. Within each stratum, an average of three sampling sites were established, with an equal number of samples drawn from different cities. Specifically, in each province/autonomous region/municipality, a provincial capital city was randomly selected as a “Class I” sampling site, representing the economic and educational center of that province/autonomous region/municipality. The other two cities were determined by considering both the geographical location of the province/autonomous region/municipality and the economic development level; one city with a moderate level of socioeconomic development was selected as a “Class II” sampling site, and another city with a relatively lower level of socioeconomic development was chosen as a “Class III” sampling site. For municipalities directly under the central government, the sample was primarily drawn using random cluster sampling, while also adhering to the principle of selecting three sampling sites per municipality. This stratified sampling approach aimed to ensure that the sample could represent the diversity of university students across different regions of China and reduce sampling bias. Through this approach, it was ensured that the survey participants adequately represented the university student population in the other areas.

#### Determination of sampling units

To ensure the representativeness and quality of the survey data, a rigorous screening mechanism was implemented during the sample selection process, wherein several universities were selected as clusters within each sampling site. Higher education institutions participating in the survey were required to possess operating qualifications recognized by the Ministry of Education, including both undergraduate and higher vocational colleges, to ensure the eligibility of the study participants. Within each university, stratified sampling was employed, with stratification based on grade level (first-year, second-year students, juniors, seniors), to ensure that the proportions of students from each grade level reflected the actual distribution within the university. The sample composition considered the reasonable distribution of demographic characteristics such as grade level and age, aiming to maintain a balanced representation of the student population. Furthermore, based on the size of the student body at each university, classes were selected proportionally as the final sampling units. Each participating university was required to assign dedicated personnel to administer the questionnaire and establish a long-term cooperative mechanism, supporting standardized and sustainable data collection. This comprehensive set of measures aimed to establish a thorough research quality control system, providing a robust data foundation for subsequent analyses.

#### Survey implementation

The survey was administered in September 2024. Electronic questionnaires were distributed uniformly via the Wenjuanxing platform, with distribution organized at the level of the administrative class group.

### Variable measurement

#### Health literacy

This study employed the Chinese version of the HLS-SF9, developed by Sun et al. (2022) based on the European Health Literacy Survey Questionnaire (HLS-EU-Q12). The scale comprises nine items that assess the ability to access, understand, evaluate, and apply health information. Scoring uses a four-point Likert scale, with options ranging from 1 = “very difficult” to 4 = “very easy,” selecting the option that best matches the participant’s situation. A higher total score indicates a higher level of health literacy. The domestically adapted HLS-SF9 has been validated and demonstrates sound psychometric properties, with an internal consistency reliability (Cronbach’s *α* coefficient) of 0.913 and a split-half reliability of 0.871. Confirmatory factor analysis showed good model fit (χ^2^/d *f* = 10.844, GFI = 0.985, AGFI = 0.971, NFI = 0.986, CFI = 0.987, RMSEA = 0.051), and no significant “ceiling effect” or “floor effect” was observed. Given the ease of administration and rapid assessment, coupled with its increasing domestic use in health literacy research and its good reliability and validity, this scale was selected as the measurement tool for health literacy in this study.

#### Life satisfaction

The SWLS, developed by Diener et al. in the 1980s (Pavot and Diener, 2009), was used to measure the overall life satisfaction of the participants. The SWLS is a well-established instrument comprising five items, utilizing a seven-point scoring standard (1 = “very dissatisfied,” 7 = “very satisfied”). Participants selected the option that best reflected their feelings, and the scores for the five items were summed. A higher total score indicates greater life satisfaction. Domestic scholars, such as Xiong and Xu (2009), have conducted reliability testing on the scale, with results showing acceptable psychometric properties, including a Cronbach’s *α* coefficient of 0.78 and a split-half reliability of 0.70. The SWLS exhibits good internal consistency and reliability, making it a suitable measure for assessing life satisfaction in the general population. The scale is structurally simple to administer, demonstrating broad international application and cross-cultural applicability. These characteristics led to the selection of this scale as the measurement tool for life satisfaction in this study.

#### Persistent exercise

This study utilized the EAS developed by Gu (2016) to evaluate the persistent exercise behavior of university students. Based on the theory of exercise behavior, the scale includes three dimensions: exercise behavior, effort input, and emotional experience, with a total of 11 items. It aims to comprehensively measure an individual’s behavioral performance, psychological effort input, and affective experience when participating in physical exercise. The scale uses a five-point Likert scale (1 = “strongly disagree,” 5 = “strongly agree”). The sum of scores for each item provides a total score, with higher scores potentially indicating a greater degree of persistent exercise. The reliability analysis results for this scale indicate high internal consistency: the Cronbach’s *α* coefficient for the total score was 0.947, and the coefficients for the three subscales were 0.875 (exercise behavior), 0.924 (effort input), and 0.897 (emotional experience). These data suggest that the scale’s reliability and validity are acceptable, potentially reflecting an individual’s characteristics of persistent exercise. This study used this scale to assess the levels of persistent exercise in university students.

#### Emotional management ability

The scale used in this study was developed by Scott et al., based on the theory proposed by Mayer and Salovey in 1990. This instrument is a representative tool in the field, containing 33 items designed to measure an individual’s ability to perceive, understand, express, regulate, and utilize their own and others’ emotions. It is divided into four dimensions, using a five-point scoring method where participants select a number corresponding to their level of agreement with each statement (1 = strongly disagree, 5 = strongly agree). Items 5, 28, and 33 are scored in reverse. Higher total scores indicate higher levels of emotional management ability. The domestic version was translated and validated by Wang (2002) at South China Normal University, with results showing an internal consistency coefficient of 0.83. The reliability of the scale has been previously demonstrated. The scale has been widely used internationally and demonstrates good reliability and validity (Schutte et al., 1998; Ciarrochi et al., 2000), suggesting it is a suitable tool for this study.

### Statistical analysis

**Figure 2:**
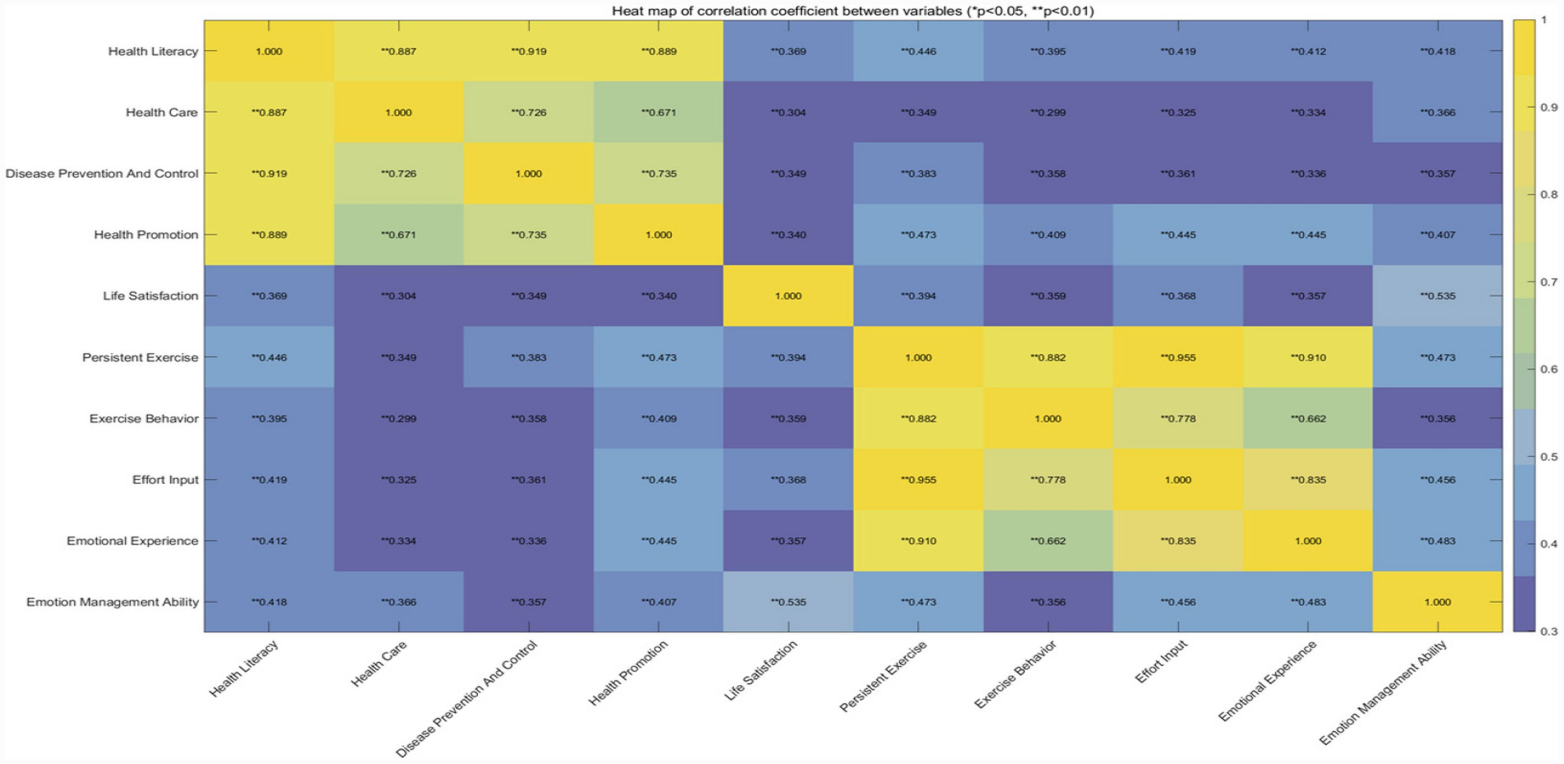
Heat map of correlation coefficient between variables. ^***^p < 0.001, ^**^p < 0.01. This heatmap displays the Pearson correlation coefficient matrix among the study variables. The intensity and gradient of colors indicate the magnitude or variation of correlation coefficients, ranging from 0.3 to 1. Refer to the color bar on the right for specific correspondences.

**Figure 3:**
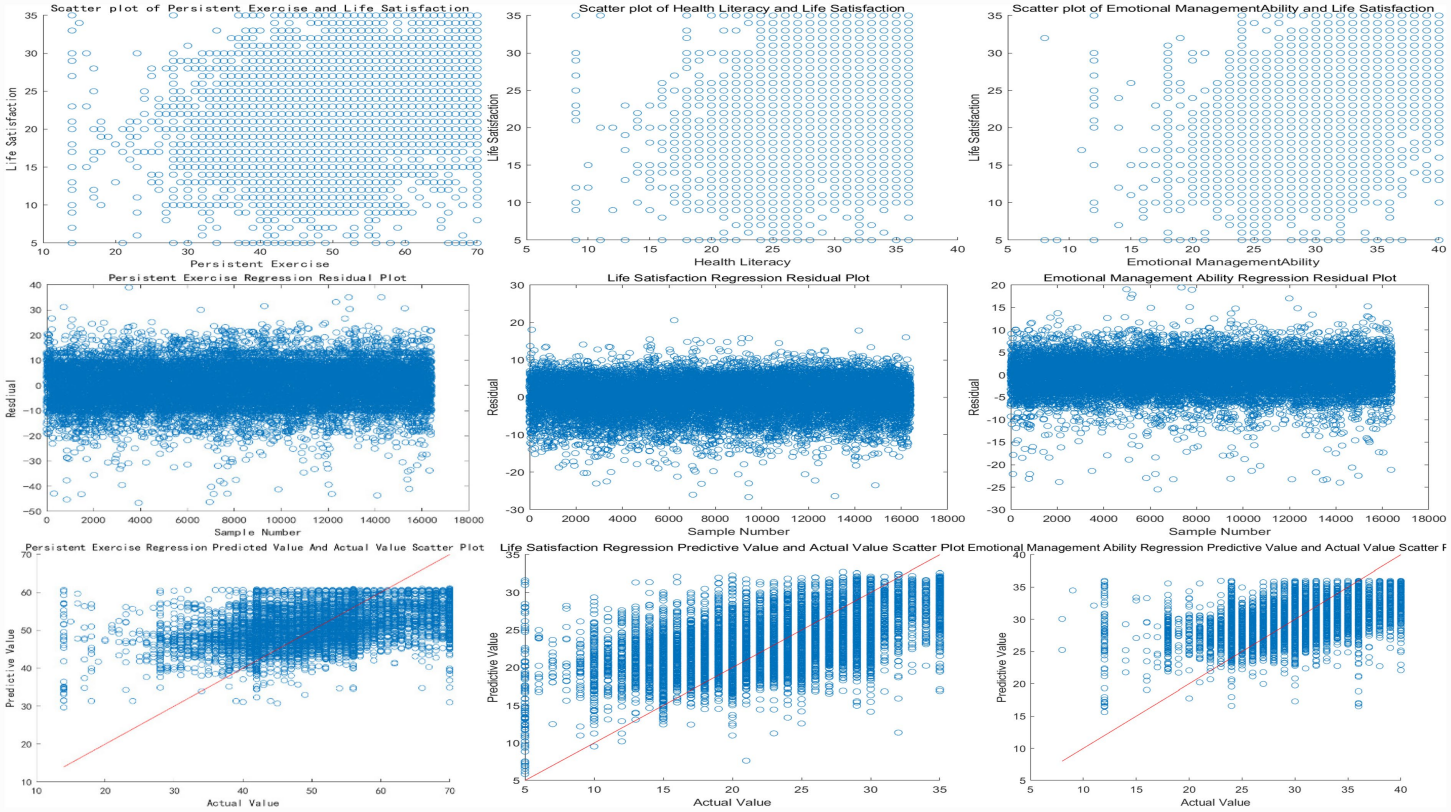
Multivariate relationship and model analysis integration diagram.

**Figure 4:**
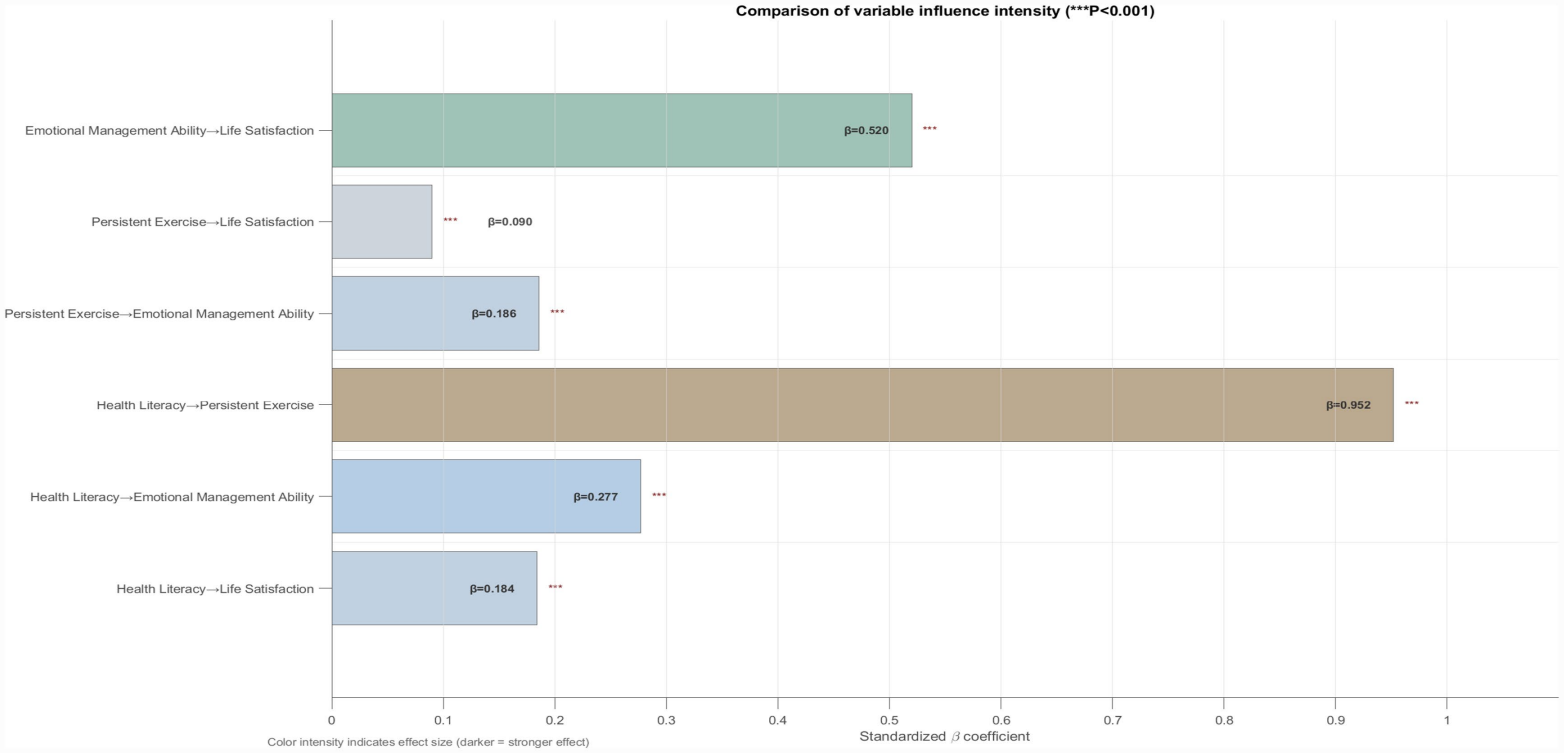
Comparison of variable influence intensity.

This study used *SPSS* 26.0 statistical software, the *PROCESS* 4.2 macro, *Microsoft Excel*, and *MATLAB* to organize and analyze the collected data. The steps included: (1) Data pre-processing, where Excel was used to perform initial processing of the data collected via the Questionnaire Star platform, and incomplete or anomalous data were either re-measured or directly removed to ensure data quality. (2) Common method variance testing, where Harman’s single-factor test was employed, with exploratory factor analysis conducted on the health literacy, life satisfaction, persistent exercise, and emotional management ability variables; the results showed that six factors with eigenvalues greater than one were extracted, with the factor with the most significant explanatory power accounting for 28.997% of the variance, a value lower than the commonly cited threshold of 40%, suggesting that standard method variance may not have been a substantial concern in the measurement data. Furthermore, we controlled for potential confounding variables in the models to further mitigate bias. (3) Normality Testing. Before parameter estimation and model fitting, normality tests were conducted for all continuous variables to ensure their normality. The skewness and kurtosis indices for each variable were calculated using SPSS 26.0 (results presented in Table 2). The absolute skewness values ranged from 0.108 to 0.284, and the absolute kurtosis values ranged from 0.163 to 1.109. Both sets of values fall well below the critical thresholds recommended by Kline (2023) (absolute skewness < 3, absolute kurtosis < 10), indicating that the data distribution adhered excellently to the normality assumption. This robust normality supports the suitability of the data for subsequent regression and structural equation modeling analyses. (4) Descriptive statistical analysis was performed to describe the basic distribution characteristics of the sample by calculating basic statistical measures such as frequencies, means, and standard deviations; the effect size *η^2^* ranged from 0 to 1, and Cohen’s interpretation standards were referenced (Zhu et al., 2024). (5) Correlation analysis, using MATLAB to generate a heatmap of inter-variable correlation coefficients (Figure 2) to visually display the strength and direction of the associations between each variable. (6) Regression analysis and mediation effect exploration, using MATLAB to visually analyze multivariate relationships (Figure 3) and assess the linearity and homoscedasticity of the relationships between variables. Additionally, to compare the relative influence strengths of the path coefficients, all continuous variables (health literacy, persistent exercise, emotional management ability, and life satisfaction) underwent z-score standardization. The variables were standardized using the z-score formula: $Z=\frac{x-\mu }{\sigma }\;$ , where *x* is the raw score, *μ* is the sample mean, and *σ* is the sample standard deviation. Following this procedure, all variables exhibited a mean of 0 and a standard deviation of 1. The standardized regression coefficients (*β*) reported in Tables 3, 4 of this study were calculated based on these standardized variables. This approach facilitates the direct comparison of path coefficients across different variables. Given the complexity of the research model and the hypothesized multiple mediation pathways, we employed two complementary analytical methods: PROCESS (Model 6) and Structural Equation Modeling (SEM). PROCESS was used for the initial exploration of chain mediation effects and the implementation of bootstrap tests. In contrast, SEM was used to verify the overall model fit and the robustness of the path coefficients. The mutual corroboration of the two methods enhances the reliability of the study’s conclusions. Regression analysis was used to explore potential mediation effects, based on the Process 4.2 macro (*Model* 6, 95% confidence interval, Bootstrap sample size of 5,000) to explore the mediating roles of persistent exercise (behavioral level) and emotional management ability (psychological level) in the relationship between health literacy and life satisfaction, with MATLAB also used to compare path coefficients and variable impact strength (Figure 4). (7) Furthermore, to control for potential confounding factors that could influence the relationship between health literacy and life satisfaction, this study included gender, grade, smoking behavior, and drinking behavior as control variables. Controlling for gender enables a more precise assessment of the direct impact of health literacy on life satisfaction, thereby mitigating the confounding effects of gender-related differences and enhancing the internal validity of the findings. By controlling for grade, we aimed to isolate the influence of health literacy on life satisfaction, thereby avoiding the potential for confounding due to differences across grade levels and increasing the accuracy of the results. Finally, controlling for smoking behavior and drinking behavior enabled us to account for the potential influence of these health behaviors on life satisfaction, thus clarifying the role of health literacy in this relationship.

**Table 2:** Normality test indicators for key variables (*N* = 16,395).

Variable	Skewness	Kurtosis
Health Literacy	0.284	1.109
Persistent Exercise	0.108	0.732
Life Satisfaction	−0.125	0.163
Emotional Management Ability	−0.137	1.029

All skewness and kurtosis values adhere to Kline’s standards for normality (Kline, 2023)(|Skewness| < 3, |Kurtosis| < 10).

**Table 3:** Regression analysis of the relationships among variables in the model.

Regression		Fitting indices			Coefficient		
Outcome variables	Predictive variables	*R*	*R* ^2^	*F*	*β*	SE	*t*
Life satisfaction		0.574	0.329	1148.573^***^			
	Health literacy				0.184	0.010	18.110^***^
	Persistent exercise				0.090	0.005	18.521^***^
	Emotional management ability				0.520	0.010	54.040^***^
	Gender				0.293	0.080	3.663^***^
	Grade				0.444	0.056	7.943^***^
	Smoking behavior				0.087	0.062	1.398
	Drinking behavior				−0.190	0.042	−4.480^***^
Emotional management ability		0.538	0.289	1112.522^***^			
	Health literacy				0.277	0.008	34.908^***^
	Persistent exercise				0.186	0.004	50.367^***^
	Gender				0.750	0.065	11.582^***^
	Grade				−0.133	0.045	−2.921
	Smoking behavior				−0.376	0.050	−7.490^***^
	Drinking behavior				−0.043	0.035	−1.246
Persistent exercise		0.483	0.233	997.237^***^			
	Health literacy				0.952	0.015	63.160^***^
	Gender				−3.546	0.134	−26.428^***^
	Grade				0.074	0.096	0.770
	Smoking behavior				0.085	0.106	0.798
	Drinking behavior				−0.355	0.073	−4.867^***^

*β*, Standardized regression coefficient; SE, Standard Error; *t*, *t*-statistic; ^***^p < 0.001, ^**^p<0.01, ^*^p < 0.05.

**Table 4:** Analysis of the mediating effects.

Variable	Name	Effect	Boot SE	LLCI	ULCI
Life satisfaction	Total effect	0.506	0.01	0.486	0.525
	Direct effect	0.183	0.01	0.164	0.203
	Indirect effect	0.322	0.008	0.306	0.338
	Health Literacy→ Persistent exercise → Life satisfaction	0.086	0.006	0.075	0.098
	Health literacy→ Emotional management ability → Life satisfaction	0.144	0.006	0.132	0.156
	Health literacy→ Persistent exercise→ Emotional management ability → Life satisfaction	0.092	0.004	0.085	0.099

Effect, Effect size; Boot SE, Bootstrap Standard Error; LLCI/ULCI, Lower/Upper Limit of the 95% Confidence Interval based on 5,000 bootstrap samples.

## Results

### Descriptive results analysis

As shown in Table 5, regarding gender differences, the total health literacy score for males (28.24 ± 4.517) was significantly higher than that for females (27.64 ± 3.832). Statistically significant intergroup differences were observed in the specific domains of health care, disease prevention and control, and health promotion (*p* < 0.001), with males exhibiting higher scores. Similarly, the total persistent exercise score and scores for each dimension were significantly higher for males (all *p* < 0.001), with the most significant difference observed in the exercise behavior dimension (*η^2^* = 0.061). Gender did not reach statistical significance in its association with life satisfaction (*p* = 0.174) or emotional management ability (*p* = 0.710). Analysis of grade level differences revealed significant variations in total health literacy scores across different grades (*p* < 0.001), with juniors leading with the highest scores (28.39 ± 4.3), which were significantly higher than those of seniors (27.36 ± 4.741). Significant grade level differences were also observed for life satisfaction, persistent exercise, and emotional management ability (*p* < 0.001). Juniors demonstrated significantly higher levels of life satisfaction (23.7 ± 6.061) and persistent exercise (51.86 ± 10.26) compared to other grades, while freshmen had the highest scores in emotional management ability (29.79 ± 4.333).

**Table 5:** Overview of descriptive analysis results (Mean ± SD).

Variable	Category	*n*	Health literacy				Life satisfaction	Persistent exercise				Emotional management ability
			Health care	Disease prevention and control	Health promotion	Total score		Exercise behavior	Effort input	Emotional experience	Total score	
Gender	Male	6,043	9.62 ± 1.616	9.18 ± 1.734	9.44 ± 1.606	28.24 ± 4.517	23.09 ± 5.935	14.7 ± 3.223	18.95 ± 3.659	19.31 ± 3.49	52.96 ± 9.677	29.69 ± 4.907
	Female	10,352	9.47 ± 1.399	8.93 ± 1.536	9.25 ± 1.378	27.64 ± 3.832	22.97 ± 5.42	13.11 ± 2.861	17.55 ± 3.282	18.3 ± 3.059	48.96 ± 8.289	29.66 ± 4.131
	Total	16,395	9.52 ± 1.484	9.02 ± 1.617	9.32 ± 1.469	27.86 ± 4.108	23.01 ± 5.616	13.7 ± 3.095	18.06 ± 3.493	18.68 ± 3.261	50.43 ± 9.034	29.67 ± 4.433
η^2^			0.002	0.006	0.004	0.005	<0.001	0.061	0.038	0.022	0.046	<0.001
*F*			38.722	97.721	63.065	80.642	1.849	1063.481	641.244	373.007	782.327	0.138
*p*			<0.001	<0.001	<0.001	<0.001	0.174	<0.001	<0.001	<0.001	<0.001	0.71
Grade	Freshman	10,170	9.53 ± 1.465	9.02 ± 1.6	9.33 ± 1.451	27.89 ± 4.037	22.83 ± 5.541	13.69 ± 2.989	18.15 ± 3.398	18.74 ± 3.177	50.58 ± 8.743	29.79 ± 4.333
	Sophomores	5,112	9.48 ± 1.504	8.99 ± 1.633	9.25 ± 1.487	27.72 ± 4.184	23.24 ± 5.663	13.63 ± 3.151	17.8 ± 3.575	18.45 ± 3.345	49.88 ± 9.278	29.36 ± 4.558
	Juniors	984	9.68 ± 1.539	9.22 ± 1.667	9.49 ± 1.497	28.39 ± 4.3	23.7 ± 6.061	14.14 ± 3.726	18.52 ± 3.848	19.2 ± 3.522	51.86 ± 10.26	30 ± 4.664
	Seniors	129	9.31 ± 1.685	8.86 ± 1.81	9.19 ± 1.767	27.36 ± 4.741	23.4 ± 5.379	13.56 ± 3.579	18.1 ± 3.974	18.78 ± 3.742	50.43 ± 10.219	29.86 ± 4.726
Total	16,395	9.52 ± 1.484	9.02 ± 1.617	9.32 ± 1.469	27.86 ± 4.108	23.01 ± 5.616	13.7 ± 3.095	18.06 ± 3.493	18.68 ± 3.261	50.43 ± 9.034	29.67 ± 4.433
η^2^			0.001	0.001	0.002	0.002	0.002	0.001	0.003	0.003	0.003	0.002
*F*			6.191	5.962	9.033	8.257	11.306	7.498	17.758	18.256	15.498	12.535
*p*			<0.001	<0.001	<0.001	<0.001	<0.001	<0.001	<0.001	<0.001	<0.001	<0.001
Smoking behavior	Never smoked	15,017	9.52 ± 1.456	9.01 ± 1.597	9.31 ± 1.445	27.83 ± 4.031	23.06 ± 5.555	13.62 ± 3.055	18.02 ± 3.443	18.65 ± 3.212	50.29 ± 8.878	29.75 ± 4.364
	Occasional smoking	1,026	9.52 ± 1.729	9.1 ± 1.802	9.34 ± 1.673	27.96 ± 4.82	22.28 ± 5.961	14.44 ± 3.247	18.42 ± 3.842	18.87 ± 3.65	51.73 ± 10.128	28.76 ± 4.876
	Frequent smoking	352	9.74 ± 1.852	9.4 ± 1.807	9.55 ± 1.778	28.68 ± 4.938	23.28 ± 6.885	14.85 ± 3.791	18.72 ± 4.303	19.41 ± 3.987	52.97 ± 11.309	29.09 ± 5.546
	Total	16,395	9.52 ± 1.484	9.02 ± 1.617	9.32 ± 1.469	27.86 ± 4.108	23.01 ± 5.616	13.7 ± 3.095	18.06 ± 3.493	18.68 ± 3.261	50.43 ± 9.034	29.67 ± 4.433
η^2^			<0.001	0.001	0.001	0.001	0.001	0.007	0.002	0.001	0.003	0.003
*F*			3.667	11.41	4.732	7.662	9.578	59.44	12.576	11.368	26.589	27.158
*p*			0.026	<0.001	0.009	<0.001	<0.001	<0.001	<0.001	<0.001	<0.001	<0.001
Drinking behavior	Never drank	7,943	9.51 ± 1.476	9.01 ± 1.615	9.28 ± 1.47	27.8 ± 4.127	23.27 ± 5.703	13.56 ± 3.048	18.03 ± 3.458	18.58 ± 3.238	50.17 ± 8.945	29.77 ± 4.566
	Drank without getting drunk	8,098	9.53 ± 1.482	9.03 ± 1.604	9.34 ± 1.452	27.9 ± 4.051	22.78 ± 5.488	13.82 ± 3.108	18.11 ± 3.49	18.78 ± 3.248	50.7 ± 9.022	29.58 ± 4.292
	Drank and got drunk	354	9.74 ± 1.683	9.12 ± 1.923	9.46 ± 1.783	28.33 ± 4.88	22.73 ± 6.276	13.84 ± 3.693	17.85 ± 4.227	18.53 ± 3.94	50.22 ± 10.949	29.46 ± 4.528
	Total	16,395	9.52 ± 1.484	9.02 ± 1.617	9.32 ± 1.469	27.86 ± 4.108	23.01 ± 5.616	13.69 ± 3.095	18.06 ± 3.493	18.67 ± 3.261	50.43 ± 9.034	29.67 ± 4.433
η^2^			0.001	<0.001	0.001	<0.001	0.002	0.002	<0.001	0.001	0.001	<0.001
*F*			4.476	0.843	5.24	3.489	15.41	13.813	1.78	7.767	7.09	3.959
*p*			0.011	0.43	0.005	0.031	<0.001	<0.001	0.169	<0.001	<0.001	0.019

*M*, mean; SD, standard deviation; *F*, *F*-statistic; *p*, *p*-value; η^2^, Partial Eta-squared.

Analysis of smoking behavior revealed statistically significant differences (*p* < 0.001) across different smoking statuses within the university student cohort in terms of disease prevention and control (a sub-dimension of health literacy), persistent exercise, life satisfaction, and emotional management ability. The data analysis results indicated that the group of university students reporting frequent smoking had the highest scores in persistent exercise, reaching 52.97 ± 11.309. Conversely, the university students who never smoked had higher scores in life satisfaction (23.06 ± 5.555) and emotional management ability (29.75 ± 4.364). Analysis of drinking behavior also showed statistically significant differences in life satisfaction, persistent exercise, and its sub-dimensions (exercise behavior and emotional experience) across different drinking statuses among university students (*p* < 0.001). The university student groups who never drank, drank without getting drunk, and drank and got drunk had the following persistent exercise scores: 50.17 ± 8.945, 50.70 ± 9.022, and 50.22 ± 10.949.

### Correlation analysis

Analysis of the data in Figure 2 reveals that disease prevention and control (*r* = 0.349) exhibited the strongest positive correlation with life satisfaction among the various sub-dimensions of health literacy. In contrast, health care (*r* = 0.304) showed a significant, but relatively weaker, positive correlation with life satisfaction. The correlation coefficient between health literacy and persistent exercise was 0.446. Among the sub-dimensions of health literacy, health promotion had the strongest positive correlation with persistent exercise, with a correlation coefficient of 0.473. The correlation coefficient between health literacy and emotional management ability was 0.418. Within the various sub-dimensions of health literacy, health promotion (*r* = 0.407) exhibited a relatively strong positive correlation with emotional management ability. The correlation coefficient between persistent exercise and life satisfaction was 0.394. Within the sub-dimensions of persistent exercise, effort input (*r* = 0.368) and emotional experience (*r* = 0.357) showed strong correlations with life satisfaction. The correlation coefficient between emotional management ability and life satisfaction was 0.535, the strongest correlation observed among all variables examined. Additionally, a significant positive correlation was found between persistent exercise and emotional management ability (*r* = 0.473).

### Regression analysis

Based on the multivariate relationship and model analysis integration diagram in Figure 3, the scatter plots of health literacy and life satisfaction, emotional management ability and life satisfaction, and persistent exercise and life satisfaction suggest a tendency for the dependent variable to increase as the independent variables increase, which is consistent with the positive correlations between variables observed in the regression analysis (Table 3), providing initial support for the relationships between variables in the model. Additionally, the residuals in the residual plots fluctuated roughly around 0 with no apparent pattern, suggesting a reasonable model fit. In the scatter plot of predicted versus actual values, the data points showed some clustering, indicating a potential predictive ability of the model. Based on these analyses, the multiple regression model used in this paper is considered reasonably adequate.

The results of the multiple regression analysis (see Table 3) indicate that, after controlling for gender, grade, smoking behavior, and drinking behavior, health literacy exhibited a significant positive association with life satisfaction (*β* = 0.184, *p* < 0.001), emotional management ability (*β* = 0.277, *p* < 0.001), and persistent exercise (*β* = 0.952, *p* < 0.001). Although the path coefficient of health literacy on persistent exercise was relatively high (*β* = 0.952, *p* < 0.001), multicollinearity diagnostics revealed that the variance inflation factor (VIF) for all predictor variables remained close to 1.000 (health literacy: VIF = 1.342; persistent exercise: VIF = 1.506; emotion management ability: VIF = 1.407; gender: VIF = 1.179; grade: VIF = 1.005; smoking behavior: VIF = 1.125; drinking behavior: VIF = 1.111). All VIF values were well below the commonly adopted threshold of 5, indicating the absence of severe multicollinearity within the model. This result suggests that, among university students, health literacy is a key driving factor for their sustained engagement in exercise. Path analysis showed that persistent exercise was significantly and positively associated with emotional management ability (*β* = 0.186, *p* < 0.001) and life satisfaction (*β* = 0.090, *p* < 0.001). Notably, emotional management ability had the strongest association with life satisfaction (*β* = 0.520, *p* < 0.001), and its explanatory power was considerably higher than that of other predictor variables. A comparison of the path variable impact strength between related variables is shown in Figure 4.

### Mediation effect test

A chain mediation effect analysis was performed using Hayes’ PROCESS 4.2 program (*Model* 6). To improve statistical power, the bias-corrected bootstrapping method was employed (with 5,000 resamples). This method aims to control for Type I errors and reduce potential estimation bias. As shown in Table 4, health literacy displayed a significant association with life satisfaction: total effect = 0.506, 95% CI [0.486, 0.525]; total direct effect = 0.183, 95% CI [0.164, 0.203]; total indirect effect = 0.322, 95% CI [0.306, 0.338]. Since none of the confidence intervals included 0, this suggests potentially significant total, direct, and indirect effects. The proportion of the total indirect effect to the total effect was 63.6%, indicating that health literacy primarily influences life satisfaction through the mediation pathways rather than through a direct path. This finding aligns with the BPSM, which emphasizes the crucial role of health behaviors and psychological resources in translating health knowledge into life satisfaction. Analyses of specific potential mediation paths revealed: (1) health literacy → persistent exercise → life satisfaction: effect = 0.086, 95% CI [0.075, 0.098]; (2) health literacy → emotional management ability → life satisfaction: effect = 0.144, 95% CI [0.132, 0.156]; (3) health literacy → persistent exercise → emotional management ability → life satisfaction: effect = 0.092, 95% CI [0.085, 0.099]. The confidence intervals for all paths did not include 0.

In addition, to provide model fit indices and more rigorously test the theoretical model, this study additionally conducted latent variable SEM analysis for the following reasons: (1) Latent variable models can separate measurement error from construct relationships, providing purer parameter estimates (Bollen, 1989); (2) Path analysis using total scores cannot provide overall model fit indices (Iacobucci, 2010); and (3) Health literacy and persistent exercise are both multi-dimensional constructs, making latent variable modeling a better fit for their theoretical structures (Edwards, 2001). However, due to the large sample size (*n* = 16,395) and the inclusion of multiple measurement indicators in the model, the χ^2^/df value was highly sensitive, resulting in a high value (121.955). This outcome is commonly observed in large-sample structural equation modeling analyses (Kline, 2023; Marsh et al., 2004; Cheung and Rensvold, 2002). Nonetheless, the CFI (0.975) and TLI (0.957) both significantly exceeded the recommended threshold of 0.90, and the RMSEA value (0.086) was close to the acceptable upper limit of 0.08. Considering all indices and accounting for the inflation effect of a large sample size on the statistic, the model fit is deemed generally acceptable. All path coefficients were statistically significant (*p* < 0.001), confirming the serial mediation effect and aligning with the conclusions of the main analysis, which further supports the hypotheses of this study. The model fit indices are presented in Table 6.

**Table 6:** Fit indices for the structural equation model.

Fit index	Expected standard	Observed value (current model)
χ^2^	—	1951.283
df	—	16
χ^2^/df	< 5	121.955
CFI	> 0.90	0.975
TLI	> 0.90	0.957
RMSEA	< 0.08	0.086 [0.083–0.089]
SRMR	< 0.08	0.284

RMSEA’s 90% confidence interval (Kline, 2023; Hu and Bentler, 1999) was [0.083, 0.089]. Although this slightly exceeds the strict standard of 0.08, it remains within an acceptable range when considering the large sample size effect. The CFI and TLI demonstrated excellent performance (both > 0.95). Based on a comprehensive assessment, the overall model fit is judged to be largely acceptable.

**Figure 5:**
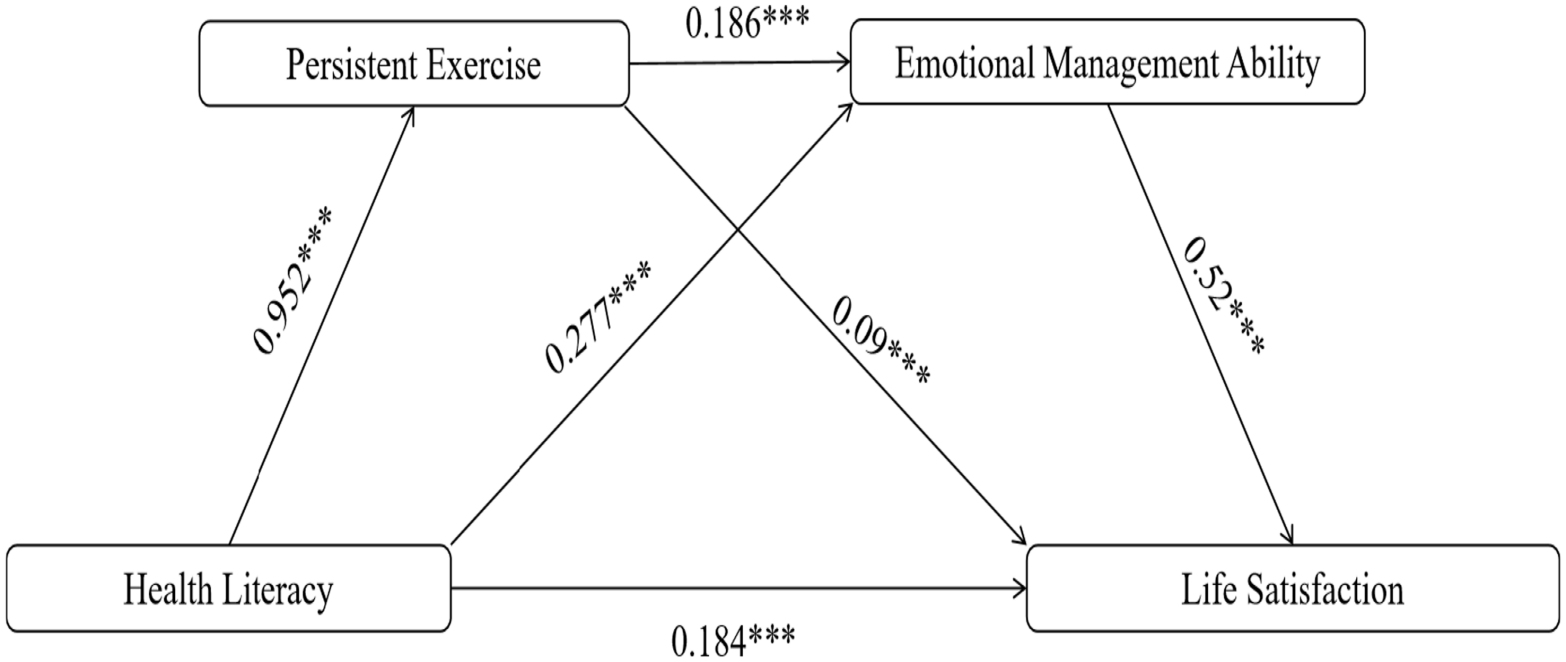
Path analysis diagram. ^***^p < 0.001, ^**^p < 0.01.

Therefore, the mediation effects of this study are illustrated in Figure 5. health literacy → persistent exercise: *β* = 0.952, *p* < 0.001; health literacy → life satisfaction: *β* = 0.184, *p* < 0.001; health literacy → emotional management ability: *β* = 0.277, *p* < 0.001; persistent exercise → emotional management ability: *β* = 0.186, *p* < 0.001; persistent exercise → life satisfaction: *β* = 0.090, *p* < 0.001; emotional management ability → life satisfaction: *β* = 0.520, *p* < 0.001. All path coefficients reached a significance level of *p* < 0.001. Bootstrapping (5,000 resamples) showed that the 95% confidence intervals for each effect value did not include 0.

## Discussion

This study systematically investigates the pathway through which health literacy is associated with life satisfaction among university students using a chained mediation model. It simultaneously examines the serial mediating effects of emotional management ability and exercise behavior. Empirical results provide support for all research hypotheses (H1-H4), suggesting that health literacy exhibits both a direct association with life satisfaction and an indirect association via the serial mediation of persistent exercise (behavioral pathway) and emotional management ability (psychological path), thereby forming a multi-level network of influence.

### The influence of health literacy on life satisfaction among Chinese university students

The research findings provide support for hypothesis 1 (H1), suggesting that health literacy is positively associated with life satisfaction in Chinese university students. Within the Chinese university student population, health literacy, as a critical ability to obtain, understand, and apply health information (Arriaga et al., 2022), exhibits a positive association that aligns with the theoretical predictions of the HBM (Von Wagner et al., 2009). This model posits that an individual’s level of health cognition plays a role in promoting exercise behavior and improving quality of life. Students possessing higher health literacy may be better equipped to accurately assess health risks, place greater emphasis on preventative health care methods, and engage in more proactive health management activities (Berkman et al., 2011). The combined effect of these abilities may manifest as higher life satisfaction and a more vibrant lived experience.

Building upon existing research, this study further elucidates the mechanisms by which health literacy influences life satisfaction through cognitive, behavioral, and psychological dimensions. Specifically, university students with higher health literacy are more likely to access reliable health information, thereby forming scientifically sound health beliefs. They are also more inclined to engage in healthy behaviors such as regular exercise and a balanced diet, leading to improved physical and mental well-being over time. Simultaneously, health literacy can enhance students’ self-efficacy, making them more confident in taking effective coping measures. On a cognitive level, students with higher health literacy may be more inclined to seek reliable health information from authoritative sources, thereby effectively avoiding the interference of misinformation and establishing sound health beliefs (Berkman et al., 2011). In terms of exercise behavior, these students may demonstrate greater enthusiasm for adopting regular exercise behavior and balanced diets, potentially leading to improvements in their physical and mental well-being through long-term accumulation (Von Wagner et al., 2009). Psychologically, enhancing one’s health literacy may boost one’s sense of self-efficacy (Bandura, 1997), potentially empowering students to confidently adopt effective coping mechanisms when facing health challenges, ultimately resulting in higher life satisfaction (Li et al., 2024). Furthermore, the various sub-dimensions of health literacy exhibit differential associations with life satisfaction, with disease prevention and control and health promotion displaying a stronger association with life satisfaction. This may be related to the specific needs of Chinese university students: the communal living environment heightens their awareness of infectious disease prevention and control (Gross, 2015; Nutbeam, 2000; Paasche-Orlow and Wolf, 2007), while academic pressures reinforce the importance of regular sleep patterns and effective stress management (Fang et al., 2024). In contrast, the health care dimension demonstrates a weaker association, possibly because basic medical knowledge is already relatively widespread among the university student population (Glover, 2022; Sakamaki et al., 2005). Cultural factors likely play a role in the association between health literacy and life satisfaction. China’s distinctive health education system emphasizes the systematic and authoritative nature of knowledge (Chen et al., 2023; Yu et al., 2007), which may promote the translation of health literacy into stable exercise behavior. Within a collectivist cultural context, the social attributes of exercise behavior (e.g., exercising with companions) may further amplify the effect of health literacy (Markus and Kitayama, 2014). Moreover, the traditional “treating disease before it manifests” preventative philosophy (Jia et al., 2020) may also reinforce the direct influence of disease prevention and control ability on life satisfaction.

The research findings suggest that health education in Chinese universities may benefit from a transition from a singular focus on knowledge dissemination toward a more practice-oriented model that emphasizes ability cultivation. Particular attention should be given to intensifying training in the assessment and application of health information for university students; much like teaching them how to “mine for gold” within a sea of information, translating scientific knowledge into practical action.

### The mediating role of persistent exercise in the relationship between health literacy and life satisfaction

The research provides support for hypothesis 2 (H2), suggesting that persistent exercise mediates the positive association between health literacy and life satisfaction in Chinese university students. Health literacy exhibits a direct association with life satisfaction and may also indirectly relate to improvements in life satisfaction by fostering persistent exercise, aligning with SCT. This theory emphasizes the dynamic interplay between individual cognition, exercise behavior, and the environment (Rovniak et al., 2002). Chinese university students’ exposure to health information (environmental factors) may influence their perceptions of exercise behavior (individual cognition), which in turn guides the formation of their exercise behavior. Persistent exercise, in turn, may relate to enhancements in their self-efficacy (individual cognition), potentially creating a cycle of “health cognition-exercise behavior-self-efficacy enhancement” (Li et al., 2024). This dynamic and interactive process may contribute to improvements in their overall quality of life.

Health literacy may relate to persistent exercise through a two-tiered pathway. On a cognitive level, when university students possess high health literacy, they may accurately understand the potential benefits of physical exercise behavior for health, such as improvements in cardiovascular function and enhanced immunity (Bennett et al., 2017), thereby stimulating intrinsic motivation for exercise behavior (Bandura, 2004). On a behavioral level, they may be better at utilizing health resources, such as fitness apps and campus facilities, to formulate scientific plans (Sun and Pan, 2025), and they may maintain exercise behavior through environmental support, such as encouragement from peers (Li et al., 2024). Ultimately, the positive emotional experiences brought about by physical exercise behavior, such as increased endorphin secretion, may further reinforce self-efficacy, forming a reciprocal relationship between cognition and exercise behavior.

The gender difference analysis reveals that males exhibit significantly greater persistent exercise compared to females. This phenomenon may be related to societal expectations of gender roles (Segar et al., 2007; Reynolds et al., 2022). Health interventions for female university students should focus on helping them overcome barriers to exercise behavior, such as creating more female-friendly exercise spaces or organizing sports events suitable for females, while also constructing more effective support environments. From a grade level perspective, juniors demonstrate extreme persistent exercise. This may be related to reduced academic pressure and increased use of campus sports resources. Universities can strengthen the cultivation of students’ exercise behavior habits in the early years to lay the foundation for long-term adherence to exercise behavior. In the descriptive analysis of this study (Table 5). However, males demonstrated higher scores in health literacy; no significant difference was observed between males and females in terms of emotional management ability. This seemingly counterintuitive finding warrants further investigation. It is plausible that the measurement of emotional management ability may not fully capture the nuanced differences in emotional expression and regulation strategies between males and females, with males potentially employing more implicit or less outwardly expressed methods to manage their emotions (Seidler et al., 2018). Alternatively, the impact of health literacy on emotional management ability may exhibit a sex-specific effect, whereby the positive influence of health literacy on emotional management is less pronounced in females than in males (Svendsen et al., 2020).

Furthermore, given the undergraduate student population involved in the study, societal and cultural expectations regarding gender roles could have, to some extent, influenced self-reports on emotional management in both sexes. Further research is required to explore these potential influencing factors. Additionally, in the descriptive analysis without controlling for other variables (Table 5), the difference in life satisfaction between genders did not reach statistical significance; however, in the regression analysis controlling for health literacy, persistent exercise, emotion management ability, grade, smoking and drinking behavior (Table 3), gender demonstrated a significant positive predictive effect on life satisfaction. This seemingly inconsistent result may reveal complex suppressor effects or confounding effects between variables (Lai et al., 2025). More specifically, certain behaviors prevalent in the male group (such as the higher observed proportions of smoking and drinking) may be linked to lower life satisfaction. These behaviors appear to “conceal” the potentially positive direct influence of gender. Once these negative behaviors are accounted for, the true predictive effect of gender emerges. This suggests that gender influences students’ life satisfaction not through a simple direct link, but via a complex interplay involving multiple health behaviors, a view consistent with the ‘person-behaviour-environment’ interaction framework of SCT (Bandura, 1986).

### The mediating role of emotional management ability in the relationship between health literacy and life satisfaction

Research hypothesis 3 (H3), which posited that emotional management ability mediates the positive association between health literacy and life satisfaction in Chinese university students, was supported by the findings. The results suggest that emotional management ability may play a mediating role between health literacy and life satisfaction. This suggests that health literacy not only exhibits a direct association with life satisfaction but may also indirectly relate to the overall life experience of university students by influencing improvements in their emotional management abilities.

Theoretical analysis suggests that health literacy may relate to emotional management ability primarily through two mechanisms. Individuals with higher health literacy may be more effective at acquiring and understanding health information related to emotional regulation, such as stress management techniques and psychological adjustment methods. This enhanced information recognition may strengthen their emotional cognitive ability (Berkman et al., 2011). University students possessing higher health literacy may be more inclined to adopt scientific strategies, such as mindfulness practice and actively seeking support, rather than resorting to harmful coping mechanisms. This may reduce the accumulation of negative emotions and enhance psychological resilience (Mann, 2004). Simultaneously, the pathway through which emotional management ability associates with life satisfaction may be multifaceted. On a physiological level, it may help maintain the balance of the neuroendocrine system, potentially reducing the negative impacts of stress on physical health (Rodrigues et al., 2020). On a psychological level, it may help maintain emotional stability under academic and employment pressures, potentially avoiding interference with cognitive function (Arsenio and Loria, 2014). On a social level, stronger emotional management abilities may be associated with harmonious relationships, which can potentially lead to increased social support (Zhu et al., 2024). These factors may contribute to increases in life satisfaction. The mediating role of emotional management ability may be particularly pronounced among Chinese university students, which may be related to the high level of competitive pressure in the higher education environment. The influence of physical health on subjective well-being may be more direct when influenced by emotional regulation (Han et al., 2022). Simultaneously, the Chinese traditional cultural emphasis on “inner restraint and balance” in emotional concepts, which may facilitate the translation of enhanced emotional management ability into increased life satisfaction (Wang, 2002), may also augment related motivations. The significance of emotional regulation in higher education deserves particular attention due to these factors. Despite males reporting higher scores in both health literacy and persistent exercise, no significant gender difference was observed in emotional regulation ability. This null finding may reflect either the limitations of the emotional regulation assessment tools in capturing gender-specific emotional expression or the influence of sociocultural factors that may suppress emotional expression in men (Wong et al., 2017).

### The serial mediating role of persistent exercise and emotional management ability

Hypothesis 4 (H4) was supported by the research findings, suggesting that the positive association between health literacy and life satisfaction in Chinese university students is serially related to persistent exercise and emotional management ability. This study suggests that health literacy has a direct association with life satisfaction. Also, it has an indirect association via the serial pathway of “health literacy → persistent exercise → emotional management ability → life satisfaction.” This finding aligns with the principles of the BPSM (Hinostroza and Mahr, 2025). Students with higher health literacy may be more likely to integrate exercise behavior into their daily routines (Sun and Pan, 2025). Persistent exercise may be related to the release of endorphins, potentially promoting physical and mental relaxation (Hinostroza and Mahr, 2025; Schuch et al., 2016) and contributing to enhancements in self-efficacy (Bernstein et al., 2020), which may empower individuals to regulate their emotions more effectively and manage academic pressures or interpersonal conflicts more effectively (Gross and John, 2003). A stable emotional state and a positive, optimistic mindset can help individuals adapt to campus life and enhance their overall life satisfaction across multiple dimensions (Gross and John, 2003). This process of interaction between physiological, psychological, and social factors may construct a health promotion chain.

This mechanism, which integrates exercise behavior changes with psychological adjustments, suggests that when designing intervention programs, we should not view persistent exercise and emotional management ability as separate entities. In programs aimed at enhancing health literacy, both professional exercise behavior guidance courses, to help students master scientific exercise behavior methods, and emotional management ability workshops, to equip students with strategies for coping with stress, should be implemented. The goal of truly enhancing students’ life satisfaction may only be achieved through such multi-layered regulation. Existing research findings can corroborate this finding: Zhu et al.’s (2024) study confirms that physical exercise behavior can indirectly improve emotional management ability by enhancing self-efficacy. Han et al. (2022) indicate that emotional regulation ability is a mediating factor between exercise behavior and mental health. The Chinese traditional cultural concept of “body and mind as one” may strengthen the association between exercise behavior and emotional state (Zhang and Xu, 2009). Within a collectivist cultural context, collective activities such as campus morning runs and sports clubs can help students develop regular exercise behavior habits and, through peer interaction and conversation, increase emotional bonds. This cultural foundation may expand the scope of the serial mediating effect. When conducting health promotion activities, universities should fully consider cultural factors and plan group health intervention programs tailored to the characteristics of Chinese university students.

The association of health literacy with life satisfaction among Chinese university students exhibits a “dual-track synchronous” characteristic, characterized by a direct association. Also, it achieves a potential influence, transforming from exercise behavior accumulation to psychological levels, along the serial pathway of “persistent exercise-emotional management ability.” This mechanism illustrates the interconnection between healthy habits and psychological well-being. Universities can use the findings of this study to integrate persistent exercise guidance and emotional management ability training into programs aimed at enhancing health literacy. At the same time, combining the Chinese traditional cultural concept of “body and mind as one” with collective activities that incorporate cultural characteristics to regulate body and mind, such as Tai Chi morning exercises and yoga meditation, can be introduced. By adopting a combination of scientific theory and cultural tradition, a health intervention program tailored to the characteristics of Chinese university students can be effectively constructed, promoting the coordinated development of students’ physical and mental well-being. Based on these findings, this study recommends that universities integrate physical activity and emotional regulation training into their health promotion programs. This could be achieved through “exercise + mindfulness” integrated courses and collective activities organized by campus sports clubs, thus enhancing students’ social support and emotional regulation abilities. Furthermore, differentiated intervention strategies should be designed for different genders and academic years. Specifically, efforts should be made to provide female students with more secure and supportive exercise environments, while introducing health behavior development programs to freshmen earlier.

### Co-occurrence of frequent smoking and persistent exercise

An unexpected finding emerged in the descriptive analysis of this study: university students who engaged in frequent smoking scored highest in adherence to exercise (Table 5). This seemingly contradictory finding is unlikely to be a mere statistical anomaly; instead, it strongly suggests the operation of a psychological mechanism known as “health compensatory beliefs” (Shuyi et al., 2025). This mechanism posits that individuals engage in a healthy behavior (such as persistent exercise) to actively or subconsciously “offset” the perceived negative health consequences associated with an unhealthy habit (such as smoking). For this cohort of undergraduates, intense physical activity may function as a psychological ‘buffer’ or ‘counterweight’, employed to alleviate the health anxiety stemming from their smoking habit. This compensation-driven behavioral pattern is likely rooted in their selective cognition regarding health risks: they may overestimate the protective efficacy of regular exercise while simultaneously downplaying the long-term, cumulative harm of smoking.

Although the sample size of this subgroup was relatively small (*n* = 352, accounting for 2.15%), it may possess specific personality traits, including a stronger propensity for sensation-seeking (Charnigo et al., 2013). This trait may manifest as both engagement in high-risk behaviors (such as smoking) and enthusiasm for high-intensity, goal-oriented activities (like persistent exercise). Alternatively, smoking and exercise might be integrated into their specific social circles within a particular social environment or peer group dynamic, which could also contribute to this correlation. This finding underscores the complexity of health behaviors, indicating that smoking status does not necessarily preclude the adoption of other health-promoting habits.

Furthermore, social desirability bias may influence self-reported outcomes (Edwards, 1958). This finding underscores the complexity of health behaviors, suggesting that in health interventions, smokers should not simply be categorized as ‘completely lacking in health awareness.’ Instead, attention should be paid to the motivational structures and psychological mechanisms underpinning their behavior. Crucially, while this finding reveals the complexity of health behavior patterns, it must be explicitly emphasized that this absolutely does not imply that the harms of smoking can be “offset” by exercise. Smoking is an unambiguous, primary health risk factor, and its negative effects are independent and profound (Murray et al., 2020). The co-occurrence observed in this study may only reflect the behavioral characteristics of a specific subgroup (such as high sensation seekers (Charnigo et al., 2013)), or a transient behavioral dynamic captured in cross-sectional data. Smoking and drinking, as powerful confounding variables, may interfere with the main mediation pathway between health literacy and life satisfaction. Therefore, interpreting this result requires extreme caution. Future research, employing longitudinal designs and qualitative interviews, is necessary to analyze the underlying motivational mechanisms and long-term health consequences deeply.

### Limitations

While this study has yielded specific, valuable findings, limitations also exist. (1) The use of a cross-sectional survey design makes it challenging to establish causal relationships between the variables of health literacy, persistent exercise, emotional management ability, and life satisfaction. It makes it challenging to track their changes over time. Moreover, this study did not conduct an in-depth analysis of the potential confounding effects of smoking and drinking behaviors within the model. Future research should adopt a longitudinal design, repeating the measurement of health literacy, persistent exercise, emotional management ability, and life satisfaction at various time points. This approach would more clearly elucidate the dynamic interaction mechanisms and causal direction among these variables. (2) Regarding sample representativeness, the survey respondents in this study primarily came from universities in Jiangsu, Northeast China, Henan, Hubei, and Guangdong, resulting in a limitation in geographical distribution. While the geographical coverage—encompassing Eastern China, the Northeast, Central China, and Southern China—partially reflects the diversity within the nation’s university student population, the sample remains heavily concentrated within provinces characterized by higher levels of economic development and comparatively superior educational resources. Crucially, this design failed to incorporate students from the Western regions, areas with relatively less developed economies, or those situated within significant ethnic minority concentrations. Furthermore, the observed relationships are potentially moderated by significant inter-regional disparities. These disparities include divergences in economic standing (Chen et al., 2021), campus athletic culture [e.g., investment in sports infrastructure (Lu, 2002), prevalence of group sporting activities (Scarapicchia et al., 2017), and curriculum design for physical education (Chen et al., 2012)], and region-specific health beliefs (Chen et al., 2020b). For instance, the economically advanced Eastern provinces may offer superior access to sporting amenities and health information pathways. In contrast, the Northeast may exhibit a distinct indoor sporting culture due to its climatic conditions. Such regional idiosyncrasies could potentially modulate the strength or the structural pathways linking health literacy, exercise persistence, and life satisfaction in this study. Consequently, caution must be exercised when extrapolating the findings of this research to the broader, national student demographic. It is important to acknowledge that the resulting sample structure exhibits certain imbalances that may influence the generalisability of the findings. Specifically, the proportion of female respondents was relatively high, accounting for 63.14% of the total, and freshmen constituted the largest cohort at 62.03%. This composition necessitates caution; for instance, observed differences in health literacy or persistent exercise between genders may exist, and first-year students, who are in an acclimatization phase of university life, may exhibit different patterns of life satisfaction or emotional regulation compared to students in their second year or above. Although the effects of both gender and year of study were statistically controlled for in the subsequent regression analyses, the inherent imbalance in the sample structure nonetheless warrants careful consideration regarding the external validity and widespread applicability of the results. The interactive effects of factors such as gender, grade, smoking behavior, and drinking behavior on the research findings were also not analyzed in depth. Future research efforts should aim to enhance the external validity of the findings by broadening the scope of sample selection. This expansion should involve incorporating student populations from underrepresented geographical areas, such as the western regions of China, and including samples from diverse types of institutions, including vocational colleges. Furthermore, subsequent studies ought to specifically investigate the moderating role of gender and year of study within the pathways influencing health literacy. The introduction of multi-group structural equation modeling would be particularly beneficial for further exploring the distinct roles played by different demographic groups within the broader mechanisms of university student health promotion. (3) This study examined the mediating effects of persistent exercise and emotional management ability. Regarding variable selection, more potential variables could be introduced, such as social support, psychological resilience, and coping styles. These variables may provide a more comprehensive explanatory mechanism for investigating the relationships between health literacy and life satisfaction. More precise measurement tools, such as accelerometers for the objective assessment of physical activity, could be used in the future to reduce the reliance on self-report exercise persistence scales and enhance the accuracy of research findings. (4) Although smoking and drinking behavior were included as control variables in the analysis, this study did not deeply explore the specific pathways through which these behaviors operate within the model, nor their interactive effects with other health behaviors and psychological states. Future research should further investigate the complex mechanisms underpinning the coexistence of health-damaging behaviors (e.g., smoking) and health-promoting behaviors (e.g., exercise), as well as their composite impact on life satisfaction. (5) The measurement of key variables, such as “exercise persistence,” relied on self-report questionnaires. Although the selected scales demonstrated satisfactory reliability and validity, the potential for social desirability bias or recall bias remains. Future studies could enhance data objectivity and precision by incorporating objective measurement tools (e.g., accelerometers, fitness trackers) to more accurately quantify physical activity.

### Practical implications

Despite the aforementioned limitations, the findings of this study offer valuable insights for university-level health education and promotion practices. The research indicates a stable association between health literacy and life satisfaction among university students, likely operating through behavioral and psychological pathways such as exercise persistence and emotional management ability. Therefore, when implementing health promotion initiatives, universities should move beyond the traditional delivery of health knowledge to focus more intently on cultivating students’ abilities to understand, evaluate, and apply health information, thereby guiding them to translate health cognition into sustainable health behaviors. Furthermore, the identified pathway linking exercise persistence to emotional management ability suggests that university health intervention programs could benefit from integrating physical activity components with psychological adjustment training. This combined approach would support the holistic physical and mental development of students. Specifically, higher education institutions should design and implement integrated ‘Exercise and Mindfulness’ blended intervention curricula. These should be systematically embedded within existing frameworks such as Compulsory Physical Education, Mental Well-being Education, or Freshman Orientation Programs, targeting the dual behavioral and psychological pathways illuminated by this study. In Physical Education modules, mindfulness breathing and emotional awareness exercises could be incorporated as structured components of warm-up or cool-down routines. Correspondingly, Mental Health curricula could integrate experiential modules centering on body–mind disciplines such as Tai Chi or Yoga. Alternatively, structured co-curricular activities, such as ‘Dawn Practice and Meditation’ group sessions organized via student societies, or targeted ‘Health Literacy Practical Workshops,’ could be established. The overarching aim of these structured curricula and activities is to facilitate the translation of health knowledge into habitual exercise behavior, while simultaneously enhancing emotional regulation capabilities during physical activity. This, in turn, is intended to foster a virtuous cycle—encompassing ‘Cognition–Behaviour–Emotion–Satisfaction’—thereby ensuring the effective conversion of health literacy into sustained life satisfaction. It must be emphasized, however, that given this study’s cross-sectional design, these practical implications are primarily inferred from the observed correlational relationships. Their actual effectiveness warrants further confirmation through future longitudinal or intervention-based research.

## Conclusion

This cross-sectional study shows there may be a significant association between health literacy and life satisfaction among Chinese university students. The study found that health literacy may be directly related to life satisfaction. Additionally, it is associated with higher life satisfaction through the mediating pathway of persistent exercise and emotional management ability. However, due to the limitations of the research design, we cannot determine the causal direction between the variables. Future research should adopt a longitudinal design to verify further the temporal mechanisms and potential causal relationships of these associations.

## Data Availability

The raw data supporting the conclusions of this article will be made available by the authors, without undue reservation.
